# Immobilization of Laccase for Oxidative Coupling of Trans-Resveratrol and Its Derivatives

**DOI:** 10.3390/ijms13055998

**Published:** 2012-05-18

**Authors:** Hong Zhang, Erna Xun, Jiaxin Wang, Ge Chen, Tiexin Cheng, Zhi Wang, Tengfei Ji, Lei Wang

**Affiliations:** 1 College of Chemistry, Jilin University, Changchun 130023, China; E-Mails: zhanghong10@jlu.edu.cn (H.Z.); ctx@jlu.edu.cn (T.C.); 2 Key Laboratory of Molecular Enzymology and Engineering of Ministry of Education, College of Life Sciences, Jilin University, Changchun 130023, China; E-Mails: xunerna86@163.com (E.X.); jiaxin1012@163.com (J.W.); chenge8lm4196@gmail.com (G.C.); 3 State Key Laboratory of Bioactive Substances and Functions of Natural Medicines, Institute of Materia Medica, Chinese Academy of Medical Sciences and Peking Union Medical College, Beijing 100050, China

**Keywords:** laccase, SBA-15, immobilization, oxidative coupling, resveratrol

## Abstract

*Trametes villosa* Laccase (TVL) was immobilized through physical adsorption on SBA-15 mesoporous silica and the immobilized TVL was used in the oxidative coupling of trans-resveratrol. Higher loading and activity of the immobilized enzyme on SBA-15 were obtained when compared with the free enzyme. The effects of reaction conditions, such as buffer type, pH, temperature and substrate concentration were investigated, and the optimum conditions were screened and resulted in enzyme activity of up to 10.3 μmol/g·h. Furthermore, the oxidative couplings of the derivatives of trans-resveratrol were also catalyzed by immobilized TVL. The immobilized TVL was recyclable and could maintain 78% of its initial activity after reusing it four times.

## 1. Introduction

Oligomeric stibenes are reported to exhibit a wide array of biological activities, such as antimicrobial, anti-HIV and antioxidant [1–3]. Resveratrol is a stilbenic phytoalexin produced by plants via a metabolic sequence induced in response to biotic or abiotic stress factors. The studies of its oligomer could provide new ideas for their pharmacological mechanisms and clinical applications [4–6]. Because the content of the oligomers in plants were too low to be obtained directly, the synthesis of the oligomers are a hot topic currently. However, only a few oligomeric resveratrols have so far been prepared in the laboratory because of their complex molecular architectures [7–9]. Since enzymatic synthesis is more efficient and highly selective when compared with the chemical synthesis, enzymatic production of structure-complex compounds has drawn great interest in recent years [10,11]. Laccases (benzenediol:oxygen oxidoreductases, EC 1.10.3.2) are multi-copper oxidases used as biocatalysts in organic synthesis, and they have great biotechnological potential due to their ability to oxidize a broad range of substrates that are employed in several industrial sectors [12,13]. Silvia reported that selective oxidative coupling of trans-resveratrol and its derivatives could be catalyzed by immobilized laccase, but the enzyme activity and reaction yield were relatively poor [14]. Therefore it is necessary to find a suitable method to increase the enzyme activity in this oxidation.

It is well known that the catalytic activity of enzyme could be even further increased by immobilization of the enzyme on a suitable support material either by covalent or noncovalent attachment [15,16]. Furthermore, the problem of recovery and reuse of enzyme with high remnant activity can also be overcome by immobilization techniques [17]. Enzyme immobilization within mesoporous materials has attracted considerable attention recently because it showed obvious advantages for both adsorption capacity and activity recovery of enzyme [18,19]. Furthermore, mesoporous materials can render the enzyme molecules more mechanically robust and stable over time. SBA-15 is a new kind of mesoporous molecular sieve. Many studies have shown that SBA-15, which possesses large surface area, high pore volume and abundant surface silanol groups is a suitable host for enzyme immobilization [20,21].

**Scheme 1:**
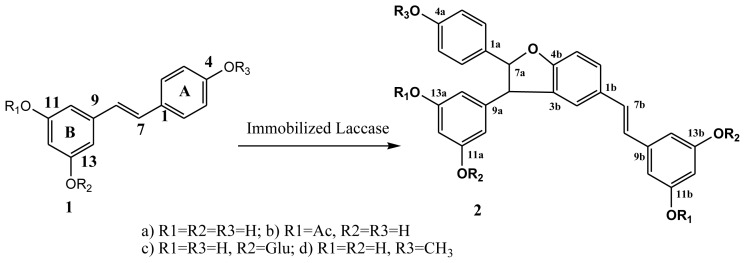
Oxidative coupling of trans-resveratrol and its derivatives catalyzed by immobilized *Trametes villosa* laccase (TVL).

In this work, we immobilized *Trametes villosa* laccase (TVL) on SBA-15 (a new kind of mesoporous molecular sieve, pore size: 10 nm) through physical adsorption. The immobilized TVL was used in oxidative coupling of trans-resveratrol as well as its derivatives (Scheme 1) and the reaction conditions were also optimized.

## 2. Results and Discussion

### 2.1. Comparison of Immobilized TVL with Free TVL

*Trametes villosa* laccase (TVL) was immobilized on SBA-15 via physical adsorption and compared with when free and on other supports (glass beads and Al_2_O_3_ beads). Table 1 showed the enzyme activity of the free and three types of immobilized TVLs under the optimum reaction conditions. The maximum immobilization amounts and enzyme activity were obtained when high-porosity SBA-15 was selected as the support, while the low-porosity aluminum oxide and glass beads yielded lower immobilization amounts and activities. The pore size of SBA-15 is suitable for that of the enzyme molecules, and the enzyme molecules could diffuse into the pores [22]. On the other hand, the activity of free laccase was decreased by degrees in aqueous media, while the terminal silanol groups present on the surface of SBA-15 may facilitate immobilization of enzymes via hydrogen bonding and enclosure of the protein in a well-defined space which may also help prevent denaturation of the protein and increase enzyme stability. Furthermore, the hydrogen bonds between the protein and the terminal silanol groups in the pores of SBA-15 could inhibit protein diffusion back out as well.

### 2.2. Effect of Buffer Type

It is well known that the type of buffer can effectively influence the enzyme activity [23]. In this study, we have investigated the effect of different buffer systems with the pH value being kept constant (pH = 5.0). As shown in Table 2, the activity of immobilized laccase was strongly influenced by the type of buffer. The interactions between enzymes and the buffer solution are complex. Specifically, an ion may affect the enzyme activity by playing the key role of a cofactor or an inhibitor. Ions may have strong interactions with the functional groups on the surface of the enzyme, especially those in the enzyme active site. This will trigger a change in the enzyme active site, both chemically and physically, thus resulting in a modification of the enzyme catalytic activity. The highest catalytic activity was obtained when the sodium citrate buffer system was used. As a result, Na_2_HPO_4_- sodium citrate buffer (pH = 5.0) was selected as the optimal media for this reaction.

### 2.3. Effect of Buffer pH

**Figure 1:**
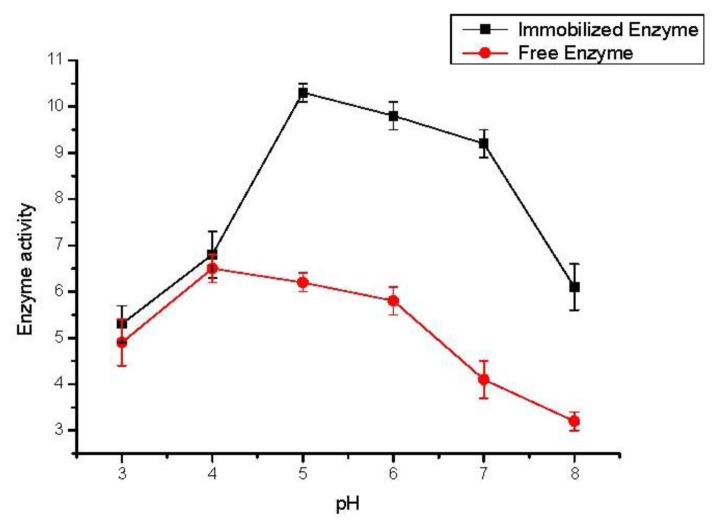
Effect of buffer pH on the activity (μmol/g·h) of free and immobilized TVL. Reaction conditions: trans-resveratrol 2 mmol, *n*-butanol 40 mL, Na2HPO4–sodium citrate buffer 10 mL (pH 3–8), immobilized TVL laccase (enzyme content: 5 mg), reaction temperature: 45 °C.

To evaluate the effect of pH on the oxidative coupling activity of immobilized TVL, the experiments were carried out with the pH values of buffer solution (Disodium hydrogen phosphate- sodium citrate buffer) between 3.0 and 8.0. The results are shown in Figure 1. The optimum pH for the oxidation by free laccase was about 4.0. The higher activity of the immobilized laccase occurred under a wider pH range of 5.0–7.0, and the optimum pH was about 5.0, which shifted into more alkaline region. It is known that the optimum pH for an immobilized enzyme shifting to a higher or lower pH depends upon surface charges of the support [24–26]. The SBA-15 has many hydroxyl groups on its inner surface, which could attract more hydrogen ions from reaction solution. It could be deduced that the pH max for the immobilized enzyme experienced a lower pH in the support pores than in the reaction media and therefore, shifted to higher pH values.

### 2.4. Effect of Temperature

**Figure 2:**
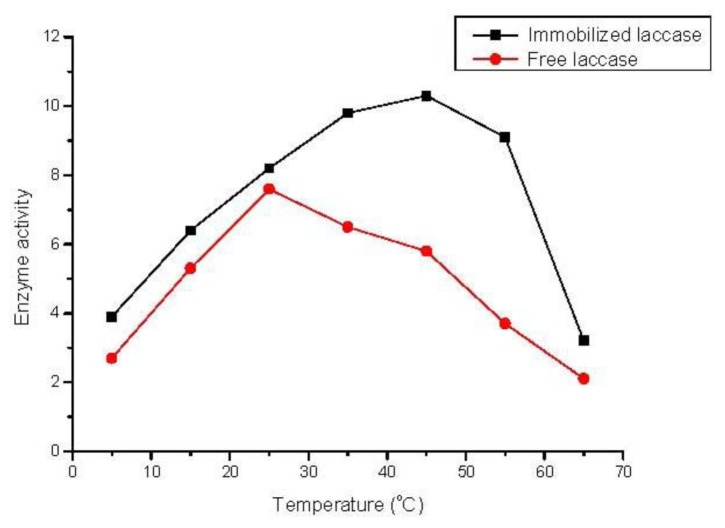
Effect of temperature on the activity (μmol/g·h) of free and immobilized TVL. Reaction conditions: trans-resveratrol 0.04 mmol/mL, *n*-butanol 40 mL, Na_2_HPO_4_–sodium citrate buffer 10 mL (pH 5.0), immobilized TVL laccase (enzyme content: 5 mg), reaction temperature: 5–65 °C

The effect of temperature on the oxidation reaction was studied over the temperature range of 5–65 °C for free and immobilized enzyme. As shown in Figure 2, either for free laccase or immobilized laccase, the initial enzymatic activity increased substantially in the lower range of temperature up to a certain point and thereafter decreased with further increases of temperature. This result was in accordance with those reported in the literature [27,28]. The optimal catalytic temperature of immobilized laccase was higher than that of free laccase. This shift for the immobilized laccase should be related to multipoint chelate interaction which caused an increase in the activation energy of the laccase to reorganize an optimum conformation for binding to its substrates. Compared with the free laccase, the immobilized laccase also exhibited a broader profile. The increased stability of the immobilized laccase was due to the restricted conformational mobility of the molecules after immobilization.

### 2.5. Effect of Substrate Concentration

**Figure 3:**
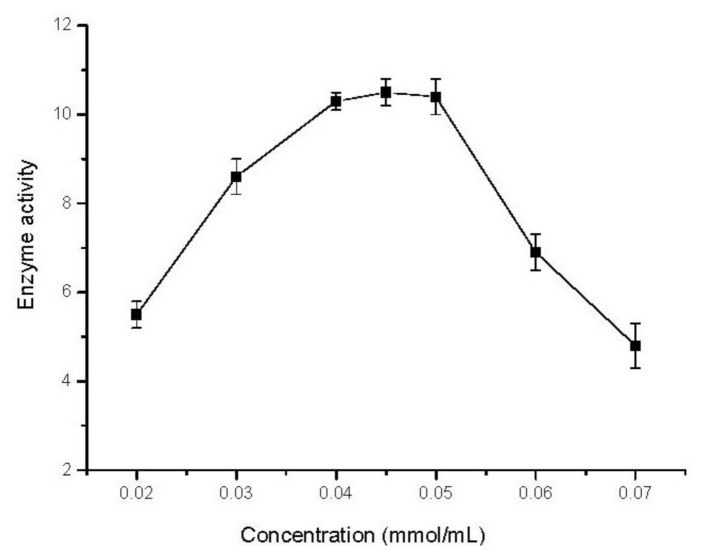
Effect of substrate concentration on the activity (μmol/g·h) of free and immobilized TVL. Reaction conditions: trans-resveratrol: 0.02–0.07 mmol, *n*-butanol 40 mL, Na_2_HPO_4_–sodium citrate buffer 10 mL; (pH 5.0), immobilized TVL laccase (enzyme content: 5 mg), reaction temperature: 45 °C

The plot in Figure 3 shows the effect of trans-resveratrol concentrations on the catalytic activity of the immobilized TVL. An increase of approximately 85% in the enzymatic activity was observed between 0.02–0.04 mmol/mL. Within the 0.04–0.05 mmol/mL range of substrate, similar activities were achieved, while the activity decreased with substrate concentrations higher than 0.05 mmol/mL. Such an observation may be attributed to the inhibition effect induced by higher concentrations of substrate [29]. Thus, the optimum substrate concentration may be considered as 0.04 mmol/mL in consideration of the atom efficiency.

### 2.6. Oxidative Coupling of Resveratrol Derivatives

To better understand the interaction of laccase with substrate and to gain more information about the properties of the products of laccase-catalyzed synthesis, the derivatives of trans-resveratrol (**1A**–**1D**) were used as substrates for the oxidation catalyzed by immoblized TVL. As shown in Table 3, the enzyme activities were obviously changed with the variation of substrate structure. It is known that laccase from *Trametes villosa* has a hydrophilic surface [30]. We suggested that the hydrophilicity of the substrate will help to form enzyme-substrate complexes and increase the reaction rate. Compound **1C**, which contains glucose is more hydrophilic than the other substrates. Therefore the highest enzyme activity was obtained when **1C** was the reaction substrate. As for compound **1D**, no reaction could be found. According to the catalytic mechanism of laccase [31], laccase could convert the 4-hydroxyl group of trans-resveratrol into an ether bond in the product, and thus this transformation would not be possible if the ether already exists prior to the reaction.

### 2.7. Reusability of Immobilized Enzyme

**Figure 4:**
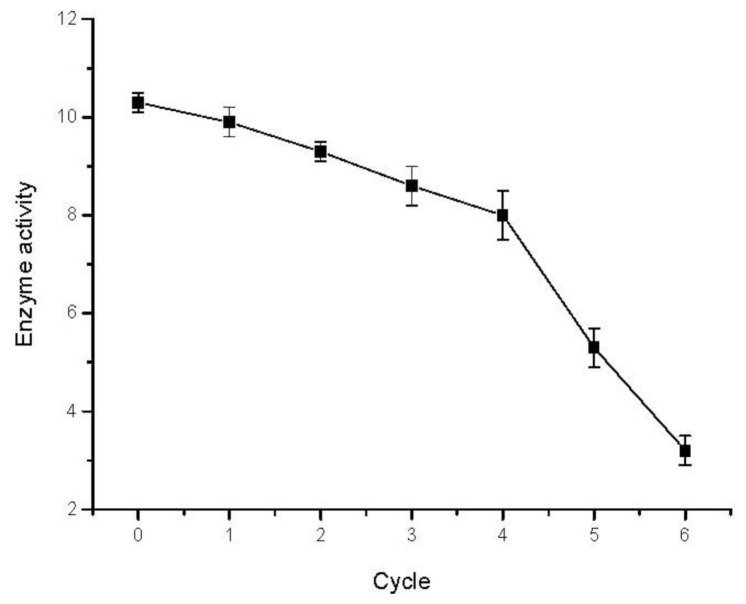
Oxidative coupling of trans-resveratrol in repeated batch process by immobilized TVL. Reaction conditions: trans-resveratrol 0.04 mmol/mL, n-butanol 40 mL, Na_2_HPO_4_–sodium citrate buffer 10 mL (pH 5.0), immobilized TVL laccase (enzyme content: 5 mg), reaction temperature: 45 °C, 48 h.

The immobilized enzyme could enhance the reusability of the enzyme compared with the free enzyme, help to cut down the production cost and make the enzymatic process economically viable [32]. The reusability of immobilized TVL was investigated in the oxidation system at 45 °C for subsequent cycles (Figure 4). The results showed that there was a gradual decrease after every cycle because of loss of a small amount of enzyme immobilized on SBA-15 or enzyme inactivation in each cycle. 78% of the initial activity of the immobilized laccase remained after 4 cycles.

## 3. Experimental Section

### 3.1. Materials

*Trametes villosa* laccase (TVL) was purchased from Sigma. Trans-resveratrol and its derivatives were isolated in our laboratory. SBA-15 (pore size: 10 nm) was kindly donated by the College of Chemistry, Jilin university and was dried at 200 °C in an oven for 5 h before use. Other reagents were all of analytical grade.

### 3.2. Immobilization of Laccase Trametes Villosa

A laccase solution (10 mL, 10 mg/mL) in sodium phosphate buffer (0.1 M, pH 8.0) was added to the tube containing the support (1 g) at 4 °C for 2 h under stirring. Then the immobilized Laccase was separated from the supernatant by centrifugation (8000 rpm) and washed with deionized water three times. The immobilized laccase was dried overnight and the amount of laccase immobilized on the support was determined using Bio-Rad DC protein assay kit (Bio-Rad, Richmond, CA, USA) with bovine serum albumin (BSA) as a standard [33] and Bound protein amount was calculated by the followed Formula:

Bound protein amount (mg/g)=amout of protein loaded/Amount of support

### 3.3. Enzymatic Oxidation of Trans-Resveratrol and Its Derivatives

Trans-resveratrol or its derivative (2 mmol) was dissolved in 40 mL *n*-butanol and 10 mL buffer solution, immobilized TVL (enzyme content: 5 mg) was added and the reaction was performed at controlled temperature. The enzyme activity (μmol/g·h) was defined as the amount (in micromoles) of trans-resveratrol converted per hour per milligram of enzyme. The reaction conversion (C) was determined by HPLC, and the structure of product was characterized by ^1^H NMR spectra and mass spectrometry, using a Varian Unity-500 spectrometer and VG AutoS12 peC-3000 mass spectrometer. The spectra are listed as follows.

2A: (+)ESI-MS *m*/*z* 477 [M + Na]^+^, (−)ESI-MS *m*/*z* 489 [M + Cl]^−; 1^H NMR (500 MHz, in CD_3_OD): δ 7.12 (2H, d, *J* = 8.0 Hz, H-2a, 6a), 6.75 (2H, d, *J* = 8.0 Hz, H-3a, 4a ), 5.35 (1H, d, *J* = 8.5 Hz, H-7a), 4.43 (1H, d, *J* = 8.5 Hz, H-8a), 6.40 (s, H-10a),6.27 (s, H-12a), 6.36(s, H-14a), 7.32 (1H, d, *J* = 8.5 Hz, H-2b), 6.80 (1H, d, *J* = 8.5 Hz, H-3b), 7.16 (s, 1H, H-6b), 6.70 (1H, d, *J* = 16.5 Hz, H-7b), 6.99 (1H, d, *J* = 16.5 Hz, H-8b), 6.73 (s, H-10b ), 6.33 (s, H-12b),6.58 (s, H-14b).

2B: (+)ESI-MS *m*/*z* 561 [M + Na]^+^, (−)ESI-MS *m*/*z* 573 [M + Cl]^−; 1^H NMR (500 MHz, in CD_3_OD): δ 7.11 (2H, d, *J* = 8.5 Hz, H-2a, 6a), 6.77 (2H, d, *J* = 8.5 Hz, H-3a, 4a ), 5.36 (1H, d, *J* = 8.0 Hz, H-7a), 4.41 (1H, d, *J* = 8.0 Hz, H-8a), 6.48 (s, H-10a),6.29 (s, H-12a), 6.33(s, H-14a), 7.31 (1H, d, *J* = 8.0 Hz, H-2b), 6.88 (1H, d, *J* = 8.0 Hz, H-3b), 7.15 (s, 1H, H-6b), 6.79(1H, d, *J* = 16.0 Hz, H-7b), 6.96 (1H, d, *J* = 16.0 Hz, H-8b), 6.71 (s, H-10b ), 6.38 (s, H-12b),6.57 (s, H-14b), 2.04 (3H, s), 2.07 (3H, s).

2C: (+)ESI-MS *m*/*z* 801 [M + Na]^+^, (−)ESI-MS *m*/*z* 813 [M + Cl]^−; 1^H NMR (500 MHz, in CD_3_OD): δ 7.11 (2H, d, *J* = 8.5 Hz, H-2a, 6a), 6.77 (2H, d, *J* = 8.5 Hz, H-3a, 4a ), 5.36 (1H, d, *J* = 8.0 Hz, H-7a), 4.41 (1H, d, *J* = 8.0 Hz, H-8a), 6.43 (s, H-10a),6.23 (s, H-12a), 6.37 (s, H-14a), 7.33 (1H, d, *J* = 8.0 Hz, H-2b), 6.81 (1H, d, *J* = 8.0 Hz, H-3b), 7.14 (s, 1H, H-6b), 6.78 (1H, d, *J* = 16.0 Hz, H-7b), 6.98 (1H, d, *J* = 16.0 Hz, H-8b), 6.71 (s, H-10b ), 6.35 (s, H-12b),6.54 (s, H-14b), 5.45 (1H, d, *J* = 8.0 Hz, H-Glc(11a), 4.42 (1H, d, *J* = 8.0 Hz, H-Glc(13b)).

### 3.4. Analytical Methods

Analysis of oxidative coupling reactions were performed at 6 h after the start of the reaction with an Agilent 1200 HPLC system on a YMC C18 column (150 mm × 4.6 mm, 5 μm) and detected at 305 nm, using methanol–H_2_O (55:45) as the mobile phase at a flow rate of 1 mL/min, sample volume: 5 μL, room temperature.

### 3.5. Reusability

To test the stability of the immobilized enzyme with repeated use, the immobilized laccase was recovered by centrifugation (3000 rpm, 15 min) after each batch and washed with buffer solution three times. Then the recycled enzyme was reused for the next batch reaction under the same conditions for 48 h.

## 4. Conclusions

In conclusion, the immobilization of TVL was successfully performed in this study, and SBA-15 showed interesting potential to be used as a support for laccase in terms of increasing the enzyme activity and stability. The immobilized laccase gave higher yields for the oxidative coupling of trans-resveratrol under the optimum reaction conditions. And the immobilized TVL also proved to be stable and retained 78% of initial activity after being reused for 4 cycles.

## Figures and Tables

**Table 1:** Protein amount and enzymatic activity of free *Trametes villosa* laccase (TVL) and TVL immobilized on different supports.

Support	Free Enzyme	Glass Beads	Al_2_O_3_	SBA-15
bound protein amount (mg/g)	-	34.9 ± 0.3	49.6 ± 0.2	95.5 ± 0.4
Enzyme activity (μmol/g·h)	5.8 ± 0.4	4.5 ± 0.3	6.2 ± 0.3	10.3 ± 0.2

Reaction conditions: trans-resveratrol 0.04 mmol/mL, *n*-butanol 40 mL, Na_2_HPO_4_–sodium citrate buffer 10 mL (pH 5.0), free or immobilized TVL laccase (enzyme content: 5 mg), reaction temperature: 45 °C

**Table 2:** Effect of buffer type on the activity of immobilized TVL.

Buffer Type	Enzyme Activity (μmol/g·h)
citric acid–sodium citrate 0.1 M	8.6 ± 0.2
Na_2_HPO_4_–sodium citrate 0.1 M	10.3 ± 0.2
NaH_2_PO_4_-Na_2_HPO_4_ 0.1 M	5.6 ± 0.4
Tris-HCl 0.1	M 6.9 ± 0.2

Reaction conditions: trans-resveratrol 0.04 mmol/mL, *n*-butanol 40 mL, buffer 10 mL (pH 5.0), immobilized TVL laccase (enzyme content: 5 mg), reaction temperature: 45 °C

**Table 3:** Effect of substrate structure on the activity of immobilized TVL.

Substrate	1A	1B	1C	1D
R_1_	H	Ac	H	H
R_2_	H	H	Glu	H
R_3_	H	H	H	CH_3_
Enzyme activity (μmol/g·h)	10.3 ± 0.2	9.5 ± 0.4	15.6 ± 0.3	0

Reaction conditions: 1A–1D 0.04 mmol/mL, *n*-butanol 40 mL, Na_2_HPO_4_–sodium citrate buffer 10 mL (pH 5.0), immobilized TVL laccase (enzyme content: 5 mg), reaction temperature: 45 °C.
